# Long-term outcomes of combination systemic and intravitreal antiviral therapy in the management of acute retinal necrosis

**DOI:** 10.1186/s12348-025-00535-0

**Published:** 2025-11-07

**Authors:** Amanda RB Wade, Zachary A. Kroeger, Sidharth K. Sengupta, Megan Guerre, Ambar Faridi, Steven Yeh, Christina J. Flaxel

**Affiliations:** 1 Casey Eye Institute at Oregon Health and Science University, 515 SW Campus Dr, Portland, OR 97239 United States; 2 Department of Ophthalmology and Visual Sciences, Stanley M. Truhlsen Eye Institute, University of Nebraska Medical Center, Omaha, NE 68105 USA

## Abstract

**Background and objective:**

To investigate whether patients diagnosed with acute retinal necrosis (ARN) previously treated with combination systemic and intravitreal antiviral injection have better long-term outcomes than those treated with systemic antiviral monotherapy

**Patients and methods:**

Follow-up retrospective cohort study analyzing data collected since 2009.^1^ Patient medical charts were reviewed for ophthalmologic data.

**Results:**

Of the original 24 patients, eight patients (nine eyes) had sufficient data for analysis. Six eyes had previously received combination systemic and intravitreal antiviral therapy, and three eyes were treated with systemic monotherapy (*P* = 0.39). Average follow-up was 177 months. Within each group, no significant difference was detected between initial and final logMAR VA (i.e. combination therapy: *P* = 0.78; systemic monotherapy: *P* = 0.60). There was no significant difference in the development of phthisis or enucleation between treatment groups.

**Conclusion:**

In this long-term cohort of ARN patients, two-thirds of patients in the original study were lost to follow-up leading to reduced sample size, however the loss was equal between the two groups. Given our limited sample size available for follow-up, there is no definitive recommendation for changing the current paradigm of treatment for this disease. Our data suggests that long-term follow-up is required for patients with ARN following their initial treatment. We recommend further studies to provide definitive recommendations in this complex patient group.

## Introduction

Acute retinal necrosis (ARN) is a rare condition characterized by anterior uveitis, vitritis, optic disc edema, occlusive vasculitis, necrotizing retinitis, and scleritis [1]. The exact incidence of ARN in the United States is unknown, but a study in the United Kingdom estimated that there is approximately one case per 1.6 to 2 million population per year [2]. Although it is relatively rare, ARN can be a severe disease, ultimately resulting in blindness in the affected eye [1]. The American Uveitis society definition of ARN is based on the syndrome’s clinical characteristics and disease course. These criteria include areas of well-demarcated peripheral retinal necrosis, rapid circumferential progression of the necrosis (in the absence of antiviral therapy), occlusive vasculopathy, and a prominent inflammatory reaction in the anterior and vitreous chambers [3]. There is evidence that members of the herpesvirus family, including varicella zoster virus (VZV), herpes simplex virus 1 and 2 (HSV-1, HSV-2), cytomegalovirus (CMV), and Epstein-Barr virus (EBV) play a causative role in the syndrome [4]. 

There are two main stages of infection in ARN. In the acute phase, the virus infects retinal cells and induces an inflammatory event that damages these cells [1]. Vaso-occlusive events may occur as vessels become inflamed, speeding necrosis of the tissue. In the late phase, contractile membranes in the vitreous and on the surface of the retina may cause breaks in the retina, especially in the areas of necrosis, which may progress to rhegmatogenous retinal detachments and permanent vision loss [1]. 

Traditionally, ARN was treated with intravenous antiviral therapy, with or without corticosteroids [5]. The goal of this treatment is to speed resolution of the disease process and to prevent contralateral eye involvement [6]. Further studies have been conducted to attempt to optimize antiviral therapy. Following several trials, newer oral agents (i.e. valacyclovir and famciclovir) demonstrated adequate vitreous penetration at concentrations high enough to inhibit replication of most VZV, HSV-1 and HSV-2 strains [6, 7]. Oral monotherapy was shown in several case studies to cause regression of retinitis as well as vision stabilization or improvement [8, 9]. 

Intravitreal injections were initially used as an adjunctive measure in severe disease to rapidly achieve therapeutic concentrations of antiviral drugs [5]. Winterhalter et al. reviewed data from the 2007 and 2012 British Ophthalmological Surveillance Unit surveys, comparing them to German data in order to analyze diagnosis, treatment, and complications in patients with ARN [10]. They found 46% of participating eye hospitals reported utilizing intravitreal injections. Outcomes related to intravitreal injections were not analyzed. Wong and colleagues performed a retrospective analysis of data collected from 1994 to 2008 from two tertiary referral centers and reported a 67% decreased risk of retinal detachment over the approximately 7-year study period in patients treated with combination systemic and intravitreal antiviral therapy [11]. This benefit was more often found in patients with moderate disease (defined as 25–50% retinal involvement) [12]. In contrast, Tibbetts and colleagues found no significant difference in outcomes (i.e. visual acuity, retinal detachment, and fellow eye involvement) between patients treated during the acyclovir-only era and patients treated during the current era of newer antiviral therapies, which include intravitreal injections [13]. Finally, Flaxel and colleagues performed a single-center retrospective cohort analysis of 24 patients, directly comparing combination intravitreal and systemic antiviral therapy to systemic therapy alone [5]. In this study, patients receiving combination therapy showed a higher incidence of greater than two-line improvement in visual acuity and a decreased incidence of retinal detachment and visual acuity loss within the first 6 months.

The purpose of this study is to investigate whether patients diagnosed with acute retinal necrosis who were previously treated with combination systemic and intravitreal antiviral injection have more favorable long-term eye health outcomes than those treated with systemic antiviral therapy alone.

## Patients and methods

This is a follow-up retrospective cohort study analyzing data collected since 2009, the cut-off for data analyzed in the original study of ARN treatment published by Flaxel and colleagues in 2013 [5]. This study was approved by the Oregon Health & Science University (OHSU) Institutional Review Board.

In the original study, 29 eyes in 24 patients were identified via a database; the full methods are described in the prior publication [5]. Inclusion criteria were patients with ARN evaluated from 1987 to 2009 at the Casey Eye Institute, Oregon Health & Science University (OHSU) Retina and Uveitis Clinics in Portland, Oregon. Patients were excluded if there was an inconclusive ARN diagnosis or less than 6 months of follow-up from initial diagnosis. If patients had lost vision in one eye, either from ARN or another cause, only the functioning eye was included in the analysis. Patients were contacted initially via phone for recruitment into this follow-up study utilizing an IRB-approved recruitment telephone script. If this was unsuccessful, or if no phone number was available, a letter was sent to their last recorded address and attempts to find updated contact information were made by contacting the referring physician’s office.

If the patient agreed to participate, they were either scheduled to be seen for an eye examination or their permission obtained for review of their medical records for ophthalmologic data, specifically Snellen visual acuity (VA) as well as presence of and time to retinal detachment, phthisis, enucleation, and involvement of the unaffected eye. These eye health outcomes were only reported if they had occurred in the interval between the original study and this follow-up study. Initial logMAR VA, defined as logMAR of the Snellen VA at the start of the original study, and final logMAR VA, defined as logMAR of the Snellen VA from the most recent eye exam, were noted. Additional demographic data (i.e., age and sex), laterality of eye involvement, and antiviral delivery route (i.e., systemic alone or a combination) were also noted. Primary outcome measures included initial and final logMAR VA, incidence of and time to retinal detachment, phthisis, and involvement of the unaffected eye. The secondary outcome measure was development of severe visual loss, defined as Snellen VA of *≤* 20/200.

Statistical analyses were conducted using Microsoft Excel (Microsoft Corporation, Redmond, Washington). Two sample T-tests were used to compare differences in mean age, length of follow-up, and initial and final logMAR VA between as well as within treatment groups, when appropriate. Fisher’s exact tests were used to compare differences in sex, number of original patients lost to follow-up, incidence of eye health outcomes between treatment groups, and to investigate the association between development of severe visual loss (VA: *≤* 20/200) and treatment group.

## Results

### Demographics

Of the original 24 patients, eight patients had data sufficient for analyses in this long-term follow-up study, either by coming in for an eye exam or agreeing to a release of medical records. These eight participants consisted of five female (62.5%) and three male patients (Table 1). Six eyes had either HSV 1 or HSV 2 by PCR testing of ocular fluids in the original study and the other eyes were only probable HSV or VZV but not confirmed. No patients were immunosuppressed nor had HIV. Five patients (six eyes) had previously received combination systemic and intravitreal antiviral therapy, and three patients (three eyes) were treated with systemic therapy alone (*P* = 0.32). Mean age with standard deviation (SD) in the combination and systemic therapy groups were 59 ± 23 years and 59 ± 31.6 years, respectively (*P* = 0.83). There were no significant differences detected in baseline patient characteristics between treatment groups for this study.

**Table 1:** Baseline characteristics of patients with ARN in the combination antiviral (systemic + antivitreal) and systemic antiviral alone treatment groups

Variable	Combination	Systemic	*P*-value
Total Patients (eyes)	5 (6)	3 (3)	0.3865
Male	2 (40%)	1 (33.3%)	
Female	3 (60%)	2 (66.7%)	0.6353
Mean age +/- SD	58.5±22.98	59±31.57	0.982

Sixteen of the original 24 patients were either unreachable (*N* = 12) or deceased (*N* = 4) and therefore lost to follow-up. The group of patients lost to follow-up included seven of the original 12 patients in the combination therapy group (58.3%) and nine of the original 12 patients in the systemic therapy alone group (75%). There was no significant difference in the number of patients lost to follow-up between treatment groups (*P* = 0.667).

## Visual acuity

Mean logMAR VA at initial presentation (prior to therapy) with standard deviation and approximate Snellen equivalent was 0.78 ± 0.6 (~ 20/125) in the combination therapy group and 1.2 ± 0.17(~ 20/320) in the systemic monotherapy group (Table 2). Final mean (± SD) logMAR VA with Snellen equivalent in the combination therapy and systemic therapy alone groups were 0.63 ± 0.1.18 (~ 20/80) and 1.63 ± 1.19 (~ 20/800), respectively. Within each treatment group, no significant difference was detected between initial and final logMAR VA (i.e. combination therapy: *P* = 0.78; systemic therapy alone: *P* = 0.60). There were also no significant differences detected in logMAR VA between treatment groups (initial logMAR VA: *P* = 0.16; final logMAR VA: *P* = 0.30).

**Table 2:** Comparison of visual acuity and incidence of other eye health outcomes in the combination antiviral (systemic + intravitreal) and systemic antiviral alone treatment

Variable	Combination	Systemic	*P*-value
Mean follow-up +/- SD (months)	165.33±16.88	200.7 +/- 25.5	0.1206
Median logMAR VA (IQR) Snellen equivalent			
Initial	0.65 (0.19-1.11) ~20/90	1.3 (1.22-1.38) ~20/400	0.1615
Final	0.14 (0-0.33) ~20/28	1.0 (0.47-1.53) ~20/200	0.2989

Initial to final P-value w/in group	0.78	0.60	
Incidence of eye health outcomes			
Enucleation	1 (20%)	0 (0%)	1
Retinal detachment	2 (40%)	2 (66.7%)	0.5238
Phthisis	1 (20%)	1 (33.3%)	1
Involvement of unaffected eye	1 (20%)	0 (0%)	1

Median logMAR VA at initial presentation (prior to therapy) with interquartile range and approximate Snellen equivalent was 0.65 (IQR 0.19–1.11; ~20/90) in the combination therapy group and 1.3 (IQR 1.22–1.38; ~20/400) in the systemic monotherapy group (Table 2). The final median logMAR VA with Snellen equivalent in the combination and systemic therapy alone groups were 0.14 (IQR 0-0.33; ~20/28) and 1.0 (IQR 0.47–1.53; ~20/200) respectively. Within each treatment group, no significant difference was detected between initial and final logMAR VA (i.e. combination therapy: *P* = 0.78; systemic therapy alone: *P* = 0.60). There were also no significant differences detected in logMAR VA between treatment groups (initial logMAR VA: *P* = 0.16; final logMAR VA: *P* = 0.30).

### Length of follow-up

Mean follow-up (± SD) for all study participants was approximately 15 years (177.1 ± 25.6 months); median follow up was 175 months. There was no significant difference in mean follow-up between treatment groups (i.e. combination therapy: 165.3 ± 16.9 months; systemic therapy alone: 200.7 ± 25.5 months; *P* = 0.12).

### Eye health outcomes

Within the combination therapy group, one patient developed phthisis approximately 11 years (131 months) after initial diagnosis and treatment, with subsequent enucleation one year later (143 months; Table 2). One patient within the combination therapy group developed contralateral involvement despite taking an appropriate prophylactic dose of oral valacyclovir. Within the systemic therapy group, two patients developed a retinal detachment. Interestingly, it was the patient without a retinal detachment in this group that developed a phthisical eye. There was no significant difference in the development of these eye health outcomes between treatment groups (incidence of phthisis: *P* = 1; incidence of enucleation: *P* = 1). Long-term treatment consisted of oral valacyclovir 1 gram daily or acyclovir 400 milligrams twice a day in all patients and instructions were given to continue indefinitely.

### Visual acuity outcomes

Throughout the duration of the study three of five patients in the combination therapy group (60%) and one patient in the systemic therapy alone group (33.3%) experienced a greater than two-line improvement in Snellen VA (*P* = 1). Additionally, one of five patients in the combination therapy group (20%) and two of the three patients in the systemic therapy alone group experienced severe visual loss, indicated by a Snellen VA *≤* 20/200. There was no noted association between treatment group and the development of severe visual loss (*P* = 0.52).

## Discussion

Since Tibbett’s study in 2010 detailing the new paradigm of treatments for acute retinal necrosis and their efficacy, much work has been done to further elucidate the role of specific therapies, particularly the addition of intravitreal injections [13]. How patients fare when comparing combined systemic and intravitreal antiviral therapies compared to systemic antiviral therapy alone has been incompletely studied. The long-term implications of ARN are unknown, including rates of recurrence, retinal detachment risk, and occurrence of phthisis. Improved understanding of this disease process would help to guide prophylactic antiviral therapy and discussions related to prognosis and disease course for patients.

In this 16-year follow-up to the original study by Flaxel and colleagues, sixteen patients involved in the original study were lost to follow-up, limiting the conclusions of this work. Of the remaining patients, six eyes received both intravitreal and oral antiviral therapy while three received oral monotherapy. The visual outcomes between initial and final logMAR VA between treatment groups were not significantly different although interestingly, the patients in the systemic antiviral alone group presented with poorer initial visual acuity and their final visual acuities were also poorer than the visual acuities observed in the combination therapy group.

Additionally, no apparent statistical significance was attributed to differences in patient characteristics; however, given the small sample size and number of patients lost to follow-up, selection bias of patients with poorer visual acuity and requiring further care may have been a factor in which patients continued to follow up. Our prior work demonstrated a reduction in retinal detachment rate and reduced incidence of severe vision loss in patients who received combination systemic and intravitreal therapy with a median follow-up time of 44 months [13]. At long-term follow-up given that retinal detachment was observed in two patients in the systemic antiviral alone group with a case of phthisis one year later, long-term monitoring and counseling patients on the risk of retinal detachment remains imperative. Given our limited sample size available for follow-up, there is no definitive recommendation for changing the current paradigm of treatment for this disease A report by the American Academy of Ophthalmology described level II and III evidence that combination systemic and intravitreal antiviral therapy may have greater therapeutic efficacy than systemic antiviral alone [4]. Our report suggests that long-term follow-up is required for patients with ARN following their initial treatment.

A single patient in this long-term cohort developed recurrence in the contralateral (left) eye. Interestingly, this same patient had two recurrences in the right eye when transitioning from valacyclovir three grams daily to one gram daily, yet the left eye activated while on full strength treatment of three grams daily. No other patients developed reactivation in either eye while on maintenance antiviral therapy. Several patients in the study had prior non-ocular herpetic manifestations without being on prophylactic anti-viral medications, and once appropriate antiviral medication was initiated, further recurrences were not observed. While documented recurrences were rare in our study, the potential for severe vision loss and the observation that one patient developed ARN in the contralateral eye and recurrent disease in the ipsilateral, affected eye is a reminder of the potential use of maintenance systemic therapy to prevent viral flares and sequelae.

Our study had several limitations, which were inherent with a long-term retrospective data analysis. Given that the data collection was retrospective, the information on ophthalmic sequelae was available through the examination findings documented and were not pre-specified. Importantly, two-thirds of patients who were a part of the original study were lost to follow-up over the current study period, leading to attrition bias. It was reassuring, however, that there was not a significant difference in the number of patients lost to follow-up in one treatment group over the other.

Historically, data suggests that with the advent of newer antivirals and PCR confirmation of a viral etiology leading to ARN may have significantly improved outcomes [13]. The management of ARN at long-term follow-up is unclear however and warrants further study. Prior studies investigating the efficacy of combination systemic and intravitreal antiviral injections in the treatment of ARN have shown promising results with favorable functional and anatomic outcomes. Preliminary long-term studies in this regard are ongoing, and a recent report by Albini showed recurrence only developed in patients who either never took or stopped oral maintenance anti-viral medications. In their series, all cases were either HSV 1 or 2, and no cases of VZV [14]. 

Our current long-term study demonstrates that following the more immediate antiviral therapy to achieve disease resolution, patients and physicians should be aware of the need for longer-term follow-up and management. Specifically, patient counseling regarding signs and symptoms of recurrence and/or retinal detachment and antiviral prophylaxis to prevent disease recurrence could be considered, particularly in patients with documented recurrences.

## Data Availability

The datasets used and/or analyzed during the current study are available from the corresponding author on reasonable request.
